# Crystallin Fusion Proteins Improve the Thermal Properties of Hair

**DOI:** 10.3389/fbioe.2019.00298

**Published:** 2019-10-25

**Authors:** Ana Tinoco, José Gonçalves, Carla Silva, Artur Cavaco-Paulo, Artur Ribeiro

**Affiliations:** Centre of Biological Engineering, University of Minho, Braga, Portugal

**Keywords:** proteins, γD-crystallin, hair, thermal damage, protection, keratin-based peptide

## Abstract

Styling hair with straightening irons is a popular daily hair routine that significantly damage the hair keratin fiber due to the high temperature applied. In this study, we investigate the effect of two fusion proteins based on the human eye γD-crystallin conjugated with a keratin-based peptide (KP-Cryst Wt and KP-Cryst Mut) on hair exposed to thermal damage. The mutant form was designed to improve protein stability and promote interaction with the hair. Through the study, it was demonstrated the protection of Asian and Caucasian virgin hair's structure by the pretreatments with the KP-Cryst fusion proteins. After hair thermal exposure, a higher water content was quantified by TGA on the hair fibers pretreated with the fusion proteins (about 38% for the KP-Cryst Wt and 44% for the KP-Cryst Mut). Also, negligible alterations in hair fibers' stiffness were observed after iron application, demonstrating the proteins capacity to effectively prevent the conversion of keratin α-helix structure into β-sheets. The results proved the capacity of the fusion proteins to bind to hair and protect it against high temperatures', supporting the development of new formulations based on the KP-Cryst proteins.

## Introduction

Human hair is a highly structured fiber organized in cuticle, cortex and sometimes medulla. The characteristics of these structures influence and determine the mechanical and optical properties of hair. For example, the stiffness of the central α-helical core of keratin and the high number of disulfide crosslinks, makes hair very resistant to external factors (Gniadecka et al., 1998).

Regardless of hair's great stability, weather, pollution, chemical treatments, and daily routine can cause several negative morphological and chemical changes in the hair (McMullen and Jachowicz, 1998; Rajput, 2015). Styling the hair with straightening irons or curling tongs is nowadays a popular daily hair routine (Christian et al., 2011), however, during these procedures, hair strands are put in contact with excessive heat (usually between 150 and 250°C) causing the disulfide bonds to break (Ettlinger et al., 2014). Both irons and curling tongs act to drive out any remaining water in the hair, promoting the formation of more bonds between hair proteins, helping to set the hair in its new conformation (Christian et al., 2011). The popularity of straightening irons and curling tongs has created a large market for hair products associated with heat styling, including heat-protection sprays, straightening balms, curl creams, and heat-protection shampoos/conditioners (Christian et al., 2011). These heat-protecting products are able to form film-like structures over the hair fibers, smoothing hair's imperfections and help protect the hair from extreme internal water loss caused by higher temperatures or long exposure time (Crudele et al., 1999).

The ability of proteins to bind to the horny layer of the hair has been explored for the development of new formulations for topical applications (Barba et al., 2010; Ribeiro et al., 2013). Proteins and peptides are considered useful for imparting gloss, softness, conditioning and manageability to the hair and prevent damage to the hair fibers, due to proteins' substantivity and their amphoteric and buffering properties (Cauwet-Martin and Dubief, 2000; Barba et al., 2010; Ribeiro et al., 2013).

Particles composed by keratin and silk fibroin were proven to recover asian hair stiffness and tensile strength while improving hair smoothness for virgin and overbleached hair (Tinoco et al., 2018). Water-soluble silk proteins, when applied on skin or hair, are able to form a durable film that improves skin and hair smoothness while protects against environmental, chemical and grooming associated damage (Fahnestock and Schultz, 2006). Milk protein hydrolysates are effective for conditioning of hair and skin and are also capable to prevent and recover the damage existent in both structures. The smoothness and softness of skin and hair are also improved when milk protein hydrolysates are applied (Tomita et al., 1994).

Crystallins are the main proteins of the vertebrate eye lens and can be divided into two major families, α-crystallins and βγ-crystallins. These proteins are able to form very stable and durable structures (Andley, 2007; Zhao et al., 2011). Human γD-crystallin is the third most abundantly expressed γ-crystallin in the lens and contains 173 amino acids (Andley, 2007). X-ray crystallographic studies showed that this monomeric protein consists of two highly homologous domains, each composed of two tightly packed β-sheet Greek key motifs that are organized as two four-stranded antiparallel β-sheets, with a total of 16 β-strands (Wistow et al., 1983; Slingsby et al., 1997). This fold, with additional contributions from domain interactions, provides this protein an unusual high level of thermodynamic stability (Zhao et al., 2011).

Previously, it was demonstrated the capacity of wild-type and mutant crystallins to bind to overbleached asian hair and improve/recover its mechanical properties. Moreover, none of the proteins displayed significant toxicity when tested in human fibroblasts (Ribeiro et al., 2013). In this work, we took advantage of the properties previously observed with the crystallin thermodynamic stability, to develop a new formulation for hair application, formulations capable to protect the keratin fibers against styling procedures using high temperatures. The new fused proteins combine the thermodynamic stability of γD-crystallin with the ability of a keratin-based peptide (KP) to bind to hair. During the design, two crystallins were used, the wild-type crystallin and a mutant crystallin. The mutant protein was constructed by the substitution of three arginine residues by three cysteine, on the wild-type crystallin sequence, that might improve the stability of proteins by the formation of covalent inner polypeptidic crosslinks (Betz, 1993). Also, the higher cysteine content of mutant crystallin promotes the protein interaction with hair keratin that is naturally reach in cysteine residues (Ribeiro et al., 2013). The KP peptide is based on the sequence of hair keratin and keratin-associated proteins, rich in cysteine residues. This cysteine residues enable the formation of intra and intermolecular disulphide bonds between the KP and the hair proteins, improving even more the capacity of crystallins to bind to the hair (Cruz et al., 2017).

In this work, a full characterization of the protective effect of KP-Cryst formulations on hair was performed by evaluating the protein ability to bind and penetrate through the hair fiber, and by measuring the effect of KP-Cryst fusion proteins on hair's mechanical properties and water content before and after iron application.

## Materials and Methods

### Materials

Natural Asian and Caucasian Brown hair samples were provided by International Hair Importers & Products Inc. (Glendale, New York, USA). The genes coding for KP-Cryst Wt and KP-Cryst Mut fusion proteins were synthesized by GenScript (New Jersey, US) and cloned in pET-28a(+) plasmid. The amino acidic sequences of KP-Cryst Wt and KP-Cryst Mut proteins are presented in Table 1. Nickel Magnetic Beads for His-Tag Protein Purification were available from GenScript, Molecular weight GRS Protein Marker Blue standards and culture medium were purchased from Grisp, Portugal. All other reagents used were of analytical grade, acquired from MerckSigma, Spain, and used as received.

**Table 1:** Amino acidic sequences of KP-Cryst Wt and KP-Cryst Mut proteins.

**Protein**	**Sequence**	**Mw (kDa)^a^**
KP-Cryst Wt	**GGVCGPSPPCITT**GAGAGAGAGAMGKITLYEDRGFQGRHYECSSDHPNLQPYLSRCNSARVDSGCWMLYEQPNYSGLQYFLRRGD YADHQQWMGLSDSVRSCRLIPHSGSHRIRLYEREDYRGQMIEFTEDCSCLQDRFRFNEIHSLNVLEGSWVLYELSNYRGRQYLLMPGD YRRYQDWGATNARVGSLRRVIDFS	24.84
KP-Cryst Mut	**GGVCGPSPPCITT**GAGAGAGAGAMGKITLYEDRGFQG$\underline{\underline{\text{C}}}$HYECSSDHPNLQPYLSRCNSA$\underline{\underline{\text{C}}}$VDSGCWMLYEQPNYSGLQYFLRR GDYADHQQWMGLSDSV$\underline{\underline{\text{C}}}$SCRLIPHSGSHRIRLYEREDYRGQMIEFTEDCSCLQDRFRFNEIHSLNVLEGSWVLYELSNYRGRQYLLM PGDYRRYQDWGATNARVGSLRRVIDFS	24.68

a *Protein final size considering the protein and the extra sequence (His-tag) from pet28a(+) vector*.

*Characters in bold correspond to the keratin-based peptide (KP) and the characters underlined correspond to the glycine-alanine linker (GA)5. The double underlined characters correspond to the substitutions of arginine residues by cysteine in the mutant form*.

### Expression and Purification of KP-Cryst Wt and KP-Cryst Mut Fusion Proteins

*Escherichia coli* BL21(DE3) containing the pET-28a(+):KP-Cryst Wt and the pET-28a(+):KP-Cryst Mut vectors were used, respectively, for KP-Cryst Wt and KP-Cryst Mut expression in Terrific Broth–Auto Induction Medium (TB-AIM). A single colony harboring the plasmid was inoculated into LB medium supplemented with kanamycin (kan) and grown overnight at 37°C. A calculated volume of the pre-inoculum was inoculated into TB-AIM_kan_ and the culture was grown for 24 h at 37°C, 200 rpm. Cells were harvested by centrifugation at 7,000 g, at 4°C, for 5 min. The cells were resuspended in phosphate buffer (20 mM sodium phosphate, 500 mM NaCl, pH 7.4) with 10 mM of imidazole and a protease inhibitor and were lysed by sonication (40% amplitude, 3.0 s on plus 9.0 s off for a total of 10 min on) in a sonicator vibra-cell^TM^ SONICS. The suspension was centrifuged at 10.000 g, for 40 min, at 4°C, to separate the soluble fraction. The protein present in the soluble fraction was purified using Nickel magnetic beads with specificity to the His-tag sequence present in the N-terminal of the proteins. Proteins purity was assessed by SDS-PAGE and the purified proteins solutions were dialyzed against distilled water for 4 days (Gonçalves et al., 2018b).

### Characterization of KP-Cryst Wt and KP-Cryst Mut Fusion Proteins

#### SDS-PAGE Analysis

Protein size and purity were analyzed by SDS-PAGE. The lyophilized proteins were solubilized in ultra-pure water, loaded on SDS-PAGE gel and stained with Coomassie Blue.

#### MALDI-TOF Mass Spectrometry

Mass/charge of KP-Cryst Wt and KP-Cryst Mut proteins was verified by MALDI-TOF using sinapic acid as the matrix (≥99.5%). The mass spectra were obtained using an Ultra-flex MALDI-TOF mass spectrophotometer (Bruker Daltonics GmbH) equipped with a 337 nm nitrogen laser. KP-Crys Wt and KP-Crys Mut proteins were detected using a double layer deposition, with a saturated solution of sinapic acid in ethanol deposited in the ground steel plate and dry. A solution of TA30 (30% acetonitrile/70% TFA) was selected to dissolve both proteins, and the resulting solution mixed in a ratio of 1:1 with a saturated solution of sinapic acid also in TA30. A sample composed by 2 μL was spotted into the ground steel target plate (Bruker part n° 209519) and analyzed using the reflective positive mode (Tinoco et al., 2019).

#### Infrared Spectroscopy (FTIR)

FTIR spectra were acquired using KBr discs made with 10 bar pressure, at room temperature, and after 16 scans with a resolution of 32 cm^−1^ from 4,000 to 600 cm^−1^ (NICOLET-AVATAR 360 FTIR spectrometer). OriginPro 8.5 software (OriginLab Corporation, MA, USA) was selected to analyze the FTIR spectra by Gaussian deconvolution of Amide I ban region (in the range of 1,600 and 1,700 cm^−1^) and conclude about the proteins secondary structure. For the Amide I deconvolution, the following procedure was performed: a linear baseline was fitted; the second derivative spectrum between 1,600 and 1,700 cm^−1^ was calculated and the secondary structure content determined considering the ratio between the areas of the assigned peak and the total area of Amide I range. Using Gaussian function, three fitting modes were tested. First, the baseline was held fixed and fitted whereas intensity and bandwidth were allowed to vary. Then, the baseline and the bandwidth were fixed and fitted again, and finally, the baseline and center peaks were fixed and fitted once more. In the end, the frequencies determined for the deconvoluted peaks were appointed to the respective protein secondary structure: β-sheet, β-turns, random coil, and α-helix (Tinoco et al., 2018).

#### Circular Dichroism (CD) Spectroscopy

The effect of temperature in the structural state of KP-Cryst Wt and KP-Cryst Mut proteins was studied by CD spectroscopy using a Jasco J-1500 spectropolarimeter equipped with a temperature controller, and a path-length cell of 1 mm. The proteins were dissolved in 5 mM potassium phosphate buffer and a concentration of 10 or 20 μM was used for all the conditions. Two different temperatures were tested in this assay: 37 and 90°C. The spectra were obtained over the wavelength interval of 180–260 nm at a scan speed of 20 nm/min and bandwidth of 1 nm (Gonçalves et al., 2018a). Relatively to the thermal scans, the monitorization of CD signal at 218 and 195 nm was performed for both proteins from 25 to 90°C, with a gradient of 3°C/min.

### Application of KP-Cryst Fusion Proteins

#### Hair Treatment With the KP-Cryst Fusion Proteins

KP-Cryst Wt and KP-Cryst Mut fusion proteins were applied to Asian and Caucasian Brown virgin hair. The hair strands were washed with a classic commercial shampoo (Pantene® Pro-V Classic) before application. For the treatments, 400 mg of each hair type were incubated with the KP-Cryst Wt and KP-Cryst Mut proteins dissolved in a buffer composed by 25 mM HEPES and 120 mM NaCl, at pH5 and pH9. Prior treatment, the hair strands were pre-conditioned with 1 mL of buffer, for 15 min at room temperature, and then dried with the hairdryer. Then, 1 mL of protein solution (1 mg/mL) was applied to the hair strands, incubated for 15 min, and dried with hairdryer. This step was repeated 5 times, with a total 5 mg of protein applied in each hair strand. Hair strands only incubated with the buffer were treated as controls. All samples were thoroughly washed in tap water with the same commercial shampoo and dried with hairdryer 24 h after treatment.

#### Substantivity Test

An amount of 20 mg of treated and untreated hair fibers were incubated with 2 mL of 1.04 × 10^−4^ M Rhodamine B aqueous solution, pH 4.1, in a bath at 50°C, for 30 min. After incubation, the fibers were rinsed 5 times with deionized water to remove loosely attached dye molecules, and were dried under a nitrogen atmosphere (dos Santos Silva and Joekes, 2005). Individual hair fibers were photographed with a fluorescence microscope (Olympus BX51, Massachusetts USA; excitation = 535/550 nm, emission = 645/675 nm) for the determination of the apparent diffusion coefficient by image analysis using image processing program ImageJ 1.46 r.

#### Differential Scanning Calorimetry (DSC)

For the DSC characterization, 2 mg of each hair samples (with and without the KP-Cryst fusion proteins) were analyzed using DSC instruments (DSC 6000, Perkin Elmer). Thermal studies of KP-Cryst Wt and KP-Cryst Mut proteins were conducted using a power compensated differential scanning calorimetry instrument and aluminum pans (max. pressure: 1 bar), at a temperature range from 50 to 265°C (heating rate: 5°C/min). The DSC instrument calibration was performed using high-purity indium and zinc and all samples were measured in duplicate, with the mean value and standard deviations calculated and presented (Tinoco et al., 2018).

#### Mechanical Properties

The effect of KP-Cryst Wt and KP-Cryst Mut proteins on the mechanical properties of hair was assessed by the differences in the Young's modulus before and after treatment with the proteins. The hair mechanical properties were determined following guidelines outlined in ASTM D1145-95 for fiber tensile testing. Tensile tests were performed using a Hounsfield dynamometer H100KS Model and a set of 25 hair fibers with low variability was selected for each condition. Each hair was individually mounted in the tensile jig by means of a paper template with a fixed gauge length of 20 mm and placed in an excicator prior to the analysis. A load range of 25 N and a speed of 1.5 mm/min were defined as settings for the tensile strength test. For each hair, applied load against extension were recorded and, using an average mean diameter of 70 μm, the data were converted to stress (load/unit area) vs. strain (% extension). All measurements were made in the middle part of the hair fiber (Tinoco et al., 2018).

### FITC Linkage to Crystallins

To study the adhesion/penetration profiles of KP-Cryst Wt and KP-Cryst Mut fusion proteins into Asian and Caucasian virgin hairs, fluorescein 5(6)-isothiocyanate (FITC) was linked to both proteins. The proteins were dissolved in 0.1 M sodium carbonate buffer, pH 9, at 2 mg/mL. A volume of 5 mL from these solutions was incubated with 250 μL of a 1 mg/mL FITC in DMSO solution, at 4°C. Unbound FITC was separated from the conjugate (KP-Cryst-FITC) by dialysis against water, at 4°C. Labeled crystallins were used in the hair treatments as described previously, in a proportion of 1:3 (KP-Cryst-FITC:KP-Cryst).

#### Confocal Microscopy

Individual hair fibers treated with the KP-Cryst-FITC conjugates were embedded in epoxy resin and transversal cuts (20 μm) were prepared using a microtome (Microtome Leitz, Oberkochen, Germany). Transversal cuts of Asian and Caucasian hairs, with and without the KP-Cryst-FITC conjugates, were analyzed by Confocal Scanning Laser Microscope (Olympus BX61, Model FluoView 1000). Acquisition for all the hair samples was done using the same settings (filter, exposure time and brightness). Images were acquired with the program FV10-Ver4.1.1.5 (Olympus) integrated with Line Series Analysis. The detection was obtained with a laser excitation line at 488 nm and emissions filters BA 505–605. Images were acquired with the program FV10-Ver4.1.1.5 (Olympus) integrated with Line Series Analysis.

### Protection Against Thermal Damage

#### Heat Application to Hair Strands Using a Flat Iron

To evaluate the protective effect of KP-Cryst proteins against heat, hair strands with and without the KP-Cryst proteins were exposed to 200°C for 5 s using a hair iron (Ricki Parodi®). This process was repeated three times, with 1 min interval between applications.

#### Mechanical Properties

The protective effect of KP-Cryst Wt and KP-Cryst Mut proteins was evaluated by the differences in the Young's modulus before and after iron application. The mechanical properties were determined using the same protocol as described in section Mechanical Properties.

#### Hair Hydration

The loss of water content from treated and untreated hair strands, before and after iron application, was evaluated using thermal gravimetric analysis (TGA). TGA was performed with a TGA 4000 (Perkin Elmer, Waltham, MA, US) using an alumina crucible with sample weights between 8-10 mg. The temperature calibration was done by Curie temperatures of reference materials: alumel, nickel and perkalloy at the same sample scanning rate. The measurements were performed from 25 to 250°C at 10°C/min under a nitrogen atmosphere (flow rate: 20 mL/min). The weight loss, in percentage, and its derivative were represented as function of temperature. The data were acquired using Pyris software (version 13).

### Statistical Analysis

Data are presented as average standard deviation (SD), *n* = 3. Statistical comparisons were performed by one-way ANOVA with GraphPad Prism 5.0 software (La Jolla, CA, USA). Tukey's *post-hoc* test was used to compare all the results between them, and a Dunnet's test was used to compare the results with a specific control. A *p*-value < 0.05 was considered to be statistically significant.

## Results and Discussion

### Characterization of KP-Cryst Wt and KP-Cryst Mut Fusion Proteins

Cosmetic industry is constantly seeking for advanced and cleaner solutions for the development of new hair care products. The approach here presented takes advantage of the ability of γD-crystallin to form film-like structures on the hair (Ribeiro et al., 2013). Exploring the great thermodynamic stability of this protein, we aim to develop new formulations to improve hair mechanical performance while protecting the hair fiber from damage resultant from the exposition to high temperatures.

Two crystallins were used in this work, the wild-type, with the same sequence as the human eye γD-crystallin, and a mutant form, obtained by the substitution of three arginine residues (R) at positions 15, 37, and 80 on crystallin sequence by cysteine residues (C). These positions, pointing toward three ‘cardinal’ positions, were selected considering previous works with mutant crystallins and amyloid-forming proteins (Meehan et al., 2004; Ribeiro et al., 2013).

The two crystallins were fused with a keratin-binding peptide (KP) (Cruz et al., 2017) and a (GA)_5_ linker (Gonçalves et al., 2018c) to promote the binding of the proteins to the hair cuticle and cortex and impart the constructs with structural mobility, respectively. The (GA)_5_ linker, composed by five repetitions of Glycine-Alanine, was included since spacers rich in glycine are described to be stable against proteolytic digestion and to impart flexibility to the proteins, being capable to bind several domains without compromising their function (Gonçalves et al., 2018c).

**Figure 1:**
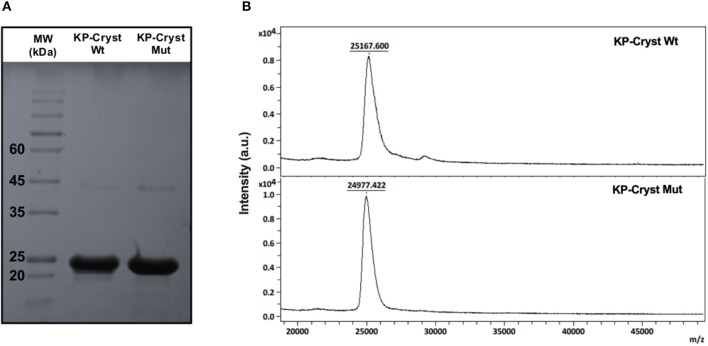
**(A)** SDS-PAGE electrophoresis of KP-Cryst-Wt and KP-Cryst-Mut fusion proteins and GRS Protein Marker Plus molecular weight; **(B)** MALDI-TOF mass spectra of KP-Cryst-Wt and KP-Cryst-Mut proteins obtained in reflective positive mode using sinapic acid as matrix.

After expression and purification, the KP-Cryst Wt and KP-Cryst Mut fusion proteins were characterized regarding purity and molecular weight. The high purity of both proteins was confirmed by SDS-PAGE and MALDI-TOF (Figure 1). On the SDS-PAGE gel (Figure 1A), it is possible to observe that both KP-Cryst Wt and KP-Cryst Mut proteins present well-defined bands with molecular weight close to the theoretical, 24.84 and 24.68 kDa, respectively. MALDI-TOF mass spectrometry (Figure 1B) also confirmed the monodisperse character and the molecular weight of KP-Cryst Wt and KP-Cryst Mut proteins.

To evaluate the effect of arginine substitutions by cysteine residues on the structure of KP-Cryst Mut variant, the FTIR spectra of KP-Cryst Wt and KP-Cryst Mut proteins were acquired. The structural information given by FTIR is predominantly derived from analysis of the so-called amide band, particularly the amide I band (1,600–1,700 cm^−1^). Deconvolution and differentiation of the amide band makes possible to distinguish between the individual component types. The deconvolution of Amide I allowed to analyze the corresponding structural assignments (Baginska et al., 2008). The components centered between 1,658 and 1,650 cm^−1^ were assigned to α-helix structures; between 1,640 and 1,620 and 1,695 and 1,690 cm^−1^ were assigned to β-sheet; and bands between 1,680 and 1,660 cm^−1^ were assigned to β-turns (Kong and Yu, 2007). The percentages corresponding to the different types of secondary structure, obtained by analysis after amide I band deconvolution, are summarized in Table 2.

**Table 2:** Secondary structural assignments on KP-Cryst Wt and KP-Cryst Mut fusion proteins.

**Secondary structure amide I**	**KP-Cryst-Wt** **%**	**KP-Cryst-Mut** **%**
β-sheet	46.29	52.72
β-Turn	22.87	17.63
Random Coil	7.6	17.45
α-helix	23.32	12.2

The substitution of arginine residues by cysteines in the KP-Cryst Mut protein resulted in an increase on the β-sheets content (from 46.29 to 52.72%) and on the amount of random coil (from 7.6 to 17.45%), when compared with the KP-Cryst Wt protein. Concomitantly, there was a reduction in the percentage of α-helix (from 23.32 to 12.2%) and of ß-turns (from 22.87 to 17.63%). Moreover, the unordered structures percentage for the KP-Cryst-Mut protein increased about 10% comparatively to the wild type protein. This increase can be attributed to an alteration in the protein structure promoted by the three mutations. Also, this effect on the protein structure could be observed in aqueous solution, where visible aggregates of KP-Cryst Mut protein have formed over time. This behavior was also observed by Ribeiro et al. (2013) for the Cryst Mut protein.

The high percentage of β-sheets on both proteins is in accordance with the literature, where is described that each domain of γ-crystallins is composed of intercalated double β-sheet Greek key motifs (Wistow et al., 1981, 1983), a characteristic structural feature of the βγ-crystallin superfamily (Aravind et al., 2009).

The structure of macromolecules, particularly proteins, is known to be sensitive to its environment, temperature and pH. The KP-Cryst fusion proteins were analyzed by circular dichroism (CD) to determine if the substitution of the arginines influenced the folding of the mutant form when comparing with the wild-type construct and to evaluate the thermal stability of the proteins at several temperatures.

**Figure 2:**
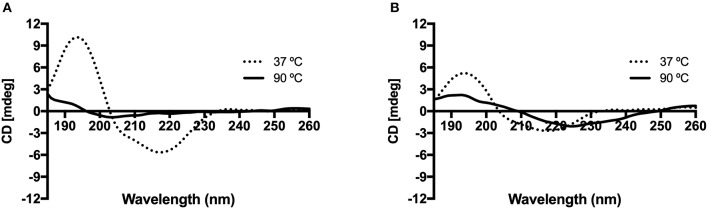
Effect of temperature on the circular dichroism spectra of KP-Cryst Wt **(A)** and KP-Cryst Mut **(B)** proteins. Experiments were conducted at 37 and 90°C.

The KP-Cryst fusion proteins were dissolved in 5 mM potassium phosphate buffer to a final concentration of 10 μM and were analyzed by CD. The CD spectra in the far-UV (below 260) was reported to predict the effect of the amino acid substitution in proteins' thermal stability (Figure 2). In Figure 2, it is possible to observe a CD spectra typical of β-sheets conformation, with a characteristic negative band near 215 and a positive band near 195 nm (Manning et al., 1988). Analyzing Figure 2, it is possible to observe, that the lower ellipticity measured for the mutant form support the effect of mutations on the proteins' structure previously observed by FTIR (Table 2).

The increasing of temperature until 90°C lead to a loss of protein conformation in both cases, however it is possible to observe that the spectrum of KP-Cryst Mut still maintain the oscillating profile observed for lower temperatures. Since the ellipticity at 90°C for the KP-Cryst Mut protein was lower than 3 mdeg, a higher concentration was tested for this protein (see Supplementary Figure 2). As expected, higher ellipticity was obtained and the same profile was observed, comparing with the lower concentration, when exposing the protein to the highest temperature tested (90°C). This result indicates that KP-Cryst Mut is more resistant to higher temperatures than the wild type protein. The increased thermal stability of KP-Cryst Mut protein was also demonstrated by the thermal scans reported at 218 and 195 nm from 25 to 90°C (see Supplementary Figure 3), where the KP-Cryst Wt protein completely denatures around 80°C while the KP-Cryst Mut protein still display some of its original structure at 90°C. Also, comparing the spectrum of both proteins at 37°C, the spectrum of KP-Crys Mut show about half of the band amplitude at 195 and 215 nm, indicating differences in the secondary structure of the protein.

The resistance to higher temperatures and the decrease in band amplitude could be due to the substitution of the three arginine residues by the cysteines in the mutant protein. This increase in protein thermal stability could also be related to the increase in protein hydrophobicity or to the formation of protein disulfide bridges resulting from the incorporation of cysteine residues in the protein sequence. It was already proved by other authors that mutations where cysteines where included, lead to the formation of disulfide bridges and, consequently, increased the proteins' thermal and structural stability (Davies and Riechmann, 1996).

### Hair Treatment With the KP-Cryst Wt and KP-Cryst Mut Fusion Proteins

To evaluate the binding capacity of KP-Cryst Wt and KP-Cryst Mut to hair, different protein solutions were prepared and applied to Asian and to Caucasian Brown virgin hairs. The proteins were dissolved at a final concentration of 1 mg/ml in 25 mM HEPES and 120 mM NaCl buffer, at pH5 or pH9. This concentration corresponds to the maximum protein solubility without the formation of visible aggregates.

The pH values were selected taking in consideration the isoelectric points of KP-Cryst Wt and KP-Cryst Mut fusion proteins, 7.13 and 6.07, respectively. Both proteins present a positive charge at pH5 and a negative charge at pH 9. Besides the effect of the presence of the KP peptide and the substitutions in the amino acidic sequence of the proteins (three extra cysteine residues for the mutant form), we also evaluated the effect of proteins' charge on their ability to bind to hair fibers.

The ability of KP-Cryst Wt and KP-Cryst Mut proteins to adhere to the hair fibers was studied using Rhodamine B as a probe. Taking in consideration the binding and diffusion of Rhodamine B into the hair, this dye gives information concerning the hair structure and the presence of polymers/proteins on the surface of the fiber (dos Santos Silva and Joekes, 2005).

**Figure 3:**
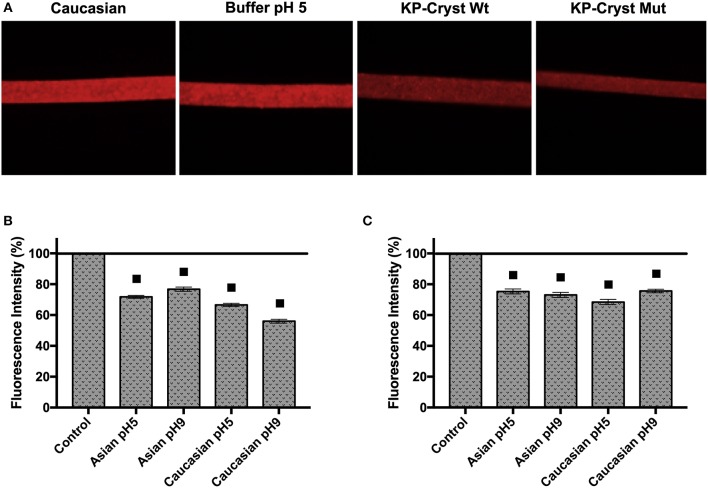
**(A)** Fluorescence microscopy images of treated and untreated Caucasian Brown hair fibers, after incubation with Rhodamine B solution; **(B)** Fibers fluorescence intensity decrease of Asian and Caucasian Brown hair after incubation with KP-Cryst Wt protein dissolved into 25 mM HEPES + 120 mM NaCl buffer, at pH 5 or pH 9; **(C)** Fibers fluorescence decrease intensity of Asian and Caucasian Brown hair after incubation with KP-Cryst Mut protein dissolved into 25 mM HEPES + 120 mM NaCl buffer at pH 5 or pH 9. Statistically significant differences from the respective control are indicated as: ■*p*-value ≤0.0001.

Images of the hair after immersion in a Rhodamine B solution, were acquired using a fluorescence microscope (Figure 3A). For each condition, 5 hair fibers were analyzed using the software ImageJ 1.50i. The fluorescence intensities were determined in three different regions for each hair fiber, and the average values were considered for the results analysis (Figures 3B,C).

The treatment with the KP-Cryst Wt and KP-Cryst Mut proteins prior to the incubation with Rhodamine B lead to a decrease in fiber's fluorescence intensity (Figure 3). The differences observed were significant when compared with the respective controls (Asian and Caucasian hairs treated with the buffers). The decrease in fluorescence was directly related with the presence of the proteins on the hair surface. Crystallins were already described to form film-like structures over the hair protecting the fiber from damage (Ribeiro et al., 2013). The reduction in fluorescence intensity might indicate that the KP-Cryst Wt and KP-Cryst Mut proteins are also able to form film-like structures over the hair cuticles, decreasing the binding and penetration degree of Rhodamine B into the hair (dos Santos Silva and Joekes, 2005).

For both proteins there was a decrease in the fluorescence intensity between 25 and 45%, with the best result obtained for the Caucasian Brown hair treated with KP-Cryst Wt at pH9. Comparing the samples treated with KP-Cryst Wt with the samples treated with KP-Cryst Mut, the most significant differences were observed for the Caucasian Brown hair at pH 9, where a difference of almost 20% was verified between the measured intensities.

Analyzing both types of hair, Asian and Caucasian Brown hair, differences in fluorescence intensities were also observed. Generally, a greater fluorescence decrease was observed for Caucasian Brown hair, when both proteins were applied. These differences could be associated with the hair structural variations like the overlapping of the cuticles on hair surface (Takahashi et al., 2006). Compared with the Asian hair, the Caucasian hair has a lower number of cuticle layers with a wider interval between them (Takahashi et al., 2006). These differences in hair structure influence protein's penetration into the hair fiber, resulting in a decrease in fiber fluorescence intensity for the Caucasian Brown hair.

**Figure 4:**
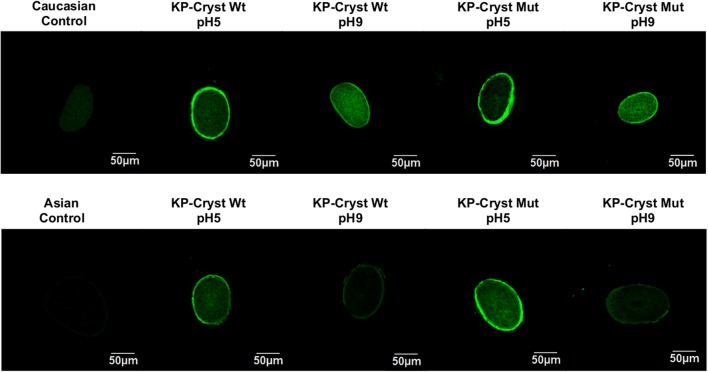
Confocal images of human hair cross sections from untreated Caucasian Brown/Asian virgin hair and Caucasian Brown/Asian virgin hair treated with KP-Cryst Wt and KP-Cryst Mut labeled with FITC, dissolved in 25 mM HEPES + 120 mM NaCl buffer, at pH 5 or pH9.

To determine the effect of the pH/protein charge and type of hair on the binding and penetration profiles of KP-Cryst Wt and KP-Cryst Mut, both proteins were conjugated with FITC and applied to Asian and Caucasian Brown virgin hairs (Figure 4).

Depending on the hair type and protein charge, the KP-Cryst Wt and KP-Crys Mut fusion proteins were able to bind to the hair cuticle and even to penetrate into the hair cortex. For the Caucasian Brown hair, the proteins bind preferentially into the hair surface/cuticle at pH5 (proteins with positive charge), while at pH9 (proteins with negative charge) they tend to penetrate into the hair cortex. This effect was expectable since higher pH values can promote the hair swelling and the raising of the cuticles, facilitating the protein penetration into the fiber cortex (Wilkerson, 1935). Also, at pH5, both proteins have a positive net charge, which can improve the proteins bounding to the negative hair surface by electrostatic interactions (Regismond et al., 1999). For the Asian hair, a different tendency was observed. A higher penetration degree and higher protein binding were obtained for pH5, regardless of the protein. This behavior could be related with structural differences between the Asian and Caucasian Brown hairs. The Asian hair has more cuticle layers, wider cuticle cells and thinner cuticular interval than the Caucasian Brown hair (Takahashi et al., 2006). These differences in the cuticle layer of the Asian hair could act as a stronger barrier, hindering the entrance of the proteins into the hair fiber cortex even at higher pH values.

### KP-Cryst Fusion Proteins Improve the Thermal Properties of Asian and Caucasian Brown Hairs

When developing a new hair care formulation, it is important to evaluate its effect on the properties of hair. DSC is a technique which can be used to evaluate the hair properties, specially to assess the interactions of the main morphological components of human hair with the components of cosmetic formulations (Barba et al., 2009). All these results can give information, for example, about the effect of new formulations on hair thermal stability. The parameter analyzed by this technique is the keratin α-helix denaturation enthalpy, which is identified by a peak with a temperature between 210 and 250°C (Monteiro et al., 2005). Peaks around these temperature range were selected to evaluate the effect of KP-Cryst Wt and KP-Cryst Mut fusion proteins on the α-helix keratin denaturation enthalpies of Asian and Caucasian Brown hair.

Analyzing the thermograms obtained by DSC (Supplementary Figure 1), two keratin denaturation peaks around 220 and 230°C were identified. This behavior was already reported by many authors, which state that these peaks correspond to the *ortho-* and *para-* cortical cells of hair, respectively. These cells are structurally different being the concentration of disulfide linkages smaller on the *ortho-*cells (Monteiro et al., 2005). Also, it is considered that the occurrence of double denaturation endotherms of keratin is attributed to the cystine content and disulfide linkages, which being large enough, make possible to separate both peaks (Monteiro et al., 2005). The enthalpy of both peaks was taken in consideration to determine the keratin α-helix denaturation enthalpies and the values are represented on Table 3.

**Table 3:** Keratin α-helix denaturation enthalpies of Caucasian Brown and Asian hair treated with KP-Cryst Wt and KP-Cryst Mut formulations, at pH5 and pH9.

**Hair**	**Condition**	**Denaturation enthalpy (J/g)**
Caucasian Brown	Control pH5	12.08 ± 0.31
	Control pH9	11.09 ± 1.09
	KP-Cryst Wt pH5	16.26 ± 0.44
	KP-Cryst Wt pH9	16.80 ± 1.15
	KP-Cryst Mut pH5	14.02 ± 1.13
	KP-Cryst Mut pH9	14.46 ± 0.46
Asian	Control pH5	14.64 ± 0.51
	Control pH9	13.16 ± 0.18
	KP-Cryst Wt pH5	14.92 ± 2.98
	KP-Cryst Wt pH9	15.64 ± 0.67
	KP-Cryst Mut pH5	14.90 ± 0.50
	KP-Cryst Mut pH9	16.73 ± 0.76

Analyzing Table 3, it was verified that both proteins, independently of the pH, are able to increase the keratin α-helix denaturation enthalpy, thus demonstrating that the proteins can interact with and stabilize the hair keratin fibrils. This increase means that more energy is need to promote keratin fibers' degradation, being the proteins able to protect the fibers against thermal damage.

When comparing the two types of hair, higher improvements were observed for the Caucasian Brown hair. These differences could be related with the structural differences between the two types of hair, with the amount of protein on the hair surface, and with the degree of penetration of the KP-Cryst Wt and KP-Cryst Mut proteins into the hair cortex. The higher number and size of the cuticles in the Asian hair might hinder the protein penetration into the fiber cortex resulting in less amount of protein available to interact with the keratin fibrils.

Comparing both proteins, KP-Cryst Wt protein increases the denaturation enthalpies 20% more than its mutant form. This may be related with the differences in the protein structure, which can disturb their interaction with the keratin filaments, or with the amount of protein present inside the hair fibers.

### Protective Effect of KP-Cryst Fusion Proteins Against Heat

Damage resulting from high temperature thermal styling treatments such as hot flat irons, blow dryers and curling irons has become an increasing concern in hair care. During these styling procedures the temperatures can exceed 200°C, imparting significant damage to the hair keratin (Zhou et al., 2011). Since these styling appliances are known to cause significant damage to hair, there is a need for the development of thermal protective formulations capable to maintain the hair properties.

The protective effect of KP-Cryst Wt and KP-Mut proteins against high temperatures was evaluated in terms of water content (TGA) and mechanical performance (stress-strain curve), before and after application of high temperatures using a hot flat iron.

**Figure 5:**
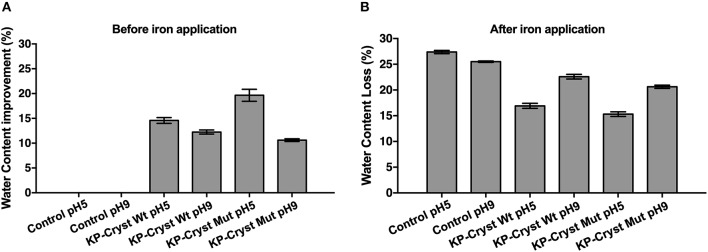
Effect of KP-Cryst Wt and KP-Cryst Mut treatment on the water content of Caucasian Brown hair fibers determined by thermal gravimetric analysis (TGA): **(A)** hair water content before iron application, with the percentages calculated relatively to the respective control; **(B)** hair water content reduction after iron application (3 × 5 s), with the percentages calculated relatively to the respective samples before iron application.

Water changes the properties of human keratin fibers and, therefore, plays an important role in cosmetic performance. Hot flat irons that lack heat control can destroy the hair protein structure resulting in changes in hair water content. Figure 5 represents the effect of KP-Cryst Wt and KP-Cryst Mut fusion proteins on hair's water content before and after iron application. The treatment with the KP-Cryst fusion proteins increased the water content of Caucasian Brown hair in more than 10% when compared with the untreated hair, with the best results being obtained for the treatments at pH5 (KP-Cryst Wt −14.58 ± 0.58%; KP-Cryst Mut −19.65 ± 1.21%). This behavior was expected since proteins show a high capacity do bind water molecules, creating a suitable environment for hair (Secchi, 2008).

After iron application (Figure 5B), a reduction of around 25% in hair's water content was verified, for both control samples. However, when the hair fibers were pretreated with the proteins, a lower reduction in the fibers' water content was obtained. The results demonstrate that the pretreatment with the KP-Cryst proteins provide a protection against heat, measured in terms of water loss resulting from the heating treatment. The percentage of heating protection was calculated based on the difference in water content reduction between the untreated hair sample and the KP-Cryst Wt or KP-Cryst Mut pretreated hair sample, after iron application. At pH5, the pretreatment with the KP-Cryst Wt and the KP-Cryst Mut proteins provided, respectively, about 38 and 44% of heating protection to the Caucasian Brown hair subjected to 200°C for 15 s (3 × 15 s). Again, the best results were verified for the treatments performed at pH5, where both proteins acquire a positive charge that improves the protein interaction with the hair's negative surface charge by electrostatic interactions (Regismond et al., 1999).

The reduction of fibers' water content after iron application can be attributed to changes in protein conformation induced by extreme heat. It is already reported in the literature that the application of heating to keratin fibers induces a change in the keratin conformation, going from an α-helix structure to β-sheets (Watt, 1975; Zhou et al., 2011). The undamaged hair displays a well-organized structure based on α-helical coiled coil protein conformation, but, once the protein is exposed to high temperatures, it can unfold and convert into a β-sheet structure. These changes in protein conformation affect the hydrogen bonding structure that stabilizes the α-helical structure and, therefore, may change the water accessibility to the hair (Zhou et al., 2011). We can deduce that, when KP-Cryst Wt and KP-Cryst Mut are applied to the hair before iron application, the proteins reduce the heat flow from the source to the hair fiber, and subsequently reduce the water loss from the hair. The protection of native keratin structure associated with the application of KP-Cryst Wt and KP-Cryst Mut proteins leads to an improved water retention, contributing to a reduction of the negative impact of heat on the hair fibers without compromising the ability of hair to be styled (data not shown).

In order to assess the protective effect of the KP-Cryst proteins in the integrity of the hair fibers, we determined the mechanical resistance of hair fibers, before and after the application of heat. Hair resistance was determined using the calculated parameters of Young's modulus (stiffness) of Caucasian Brown hair treated with both wild-type and mutant proteins, before and after iron application (3 × 5 s, 200°C). The protective effect of the proteins could be determined taking in consideration these two parameters, since the slightest modification in the chemical composition or in the structure of hair may greatly alter its mechanical properties (Ribeiro et al., 2013).

**Figure 6:**
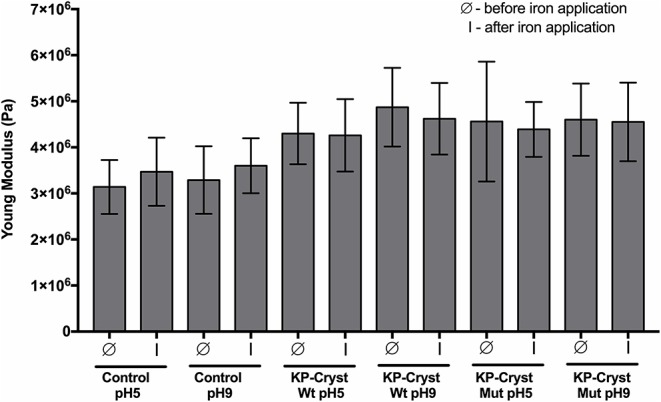
Mechanical resistance parameters: Young's modulus of Caucasian Brown hair before (Ø) and after (I) iron application (3 × 5 s). Values are the mean ± SD of 30 independent measurements.

After proving the affinity of both proteins toward the Caucasian Brown hair, our objective was to check their ability to improve the mechanical properties of Caucasian Brown hair and protect it against heating procedures that thermally damage hairs' keratinous structure (Figure 6).

Both proteins, independently of pH, were able to significantly improve the Caucasian Brown hair's Young's modulus (*p*-value < 0.0001) after treatment (Figure 6). The application of KP-Cryst Wt protein resulted in an improvement of hair's Young's modulus in a magnitude of 37 and 48% when applied at pH5 and at pH9, respectively. Relatively to the KP-Cryst Mut protein, the improvement was around 45 and 40% when applied at pH5 and at pH9, respectively. The improvement in the Young's modulus after proteins' application could be related with the interaction between these proteins with the keratin fibers, resulting in an increase in fibers' stiffness. Also, the capacity of these proteins to penetrate into the fiber cortex, demonstrated on Figure 4, seems to be an essential condition for the improvement of hair's mechanical properties. The increase in Young's modulus could also be related with the KP-Cryst Wt and KP-Cryst Mut structure. Proteins structurally characterized by the Greek key motifs show the ability to coat specific structures, so these proteins might form a thin coating around the hair fibers (Wistow et al., 1985; Ribeiro et al., 2013), decreasing the hair elasticity and, in consequent, increasing the Young's modulus values. No significant differences were observed between the hair fibers treated with KP-Cryst Wt and KP-Cryst Mut proteins, regardless of the pH tested.

After iron application, a 10% increase in the Young's modulus values was observed for the control samples, comparatively to the values obtained before iron application. This increase could be attributed to the modifications of hair proteins induced by excessive heating, including the conversion of keratin structure from α-helix to β-sheet. Some authors propose that the conversion of α-helix into β-sheet is driven by the disruption of the hydrogen bonding structure that hold together the protein helices, suggesting the unfolding of part of the helical structure and concomitant refolding into β-sheet (Zhou et al., 2011). This change into a keratin β-sheets structure increases the fibers' crystallinity, resulting in an increase of their stiffness.

When the KP-Cryst Wt and KP-Cryst Mut were applied on the Caucasian Brown hair as a pretreatment, a smaller change in the Young's modulus value was verified after exposition to high temperatures, when compared to the values before iron application. These results support the protective role of KP-Cryst proteins, since the pretreatment with four protein-based formulations effectively prevented the conversion of keratin α-helix structure into β-sheets, resulting in negligible alteration in the hair fibers' stiffness values (Zhou et al., 2011). One may assume that the protection against high temperatures can be associated with the structure of these two proteins which are composed by a pair of domains containing two tightly packed Greek key β-sheets motif. This fold, in conjugation with contributions from domain interactions, contributes toward an unusually high thermodynamic stability of crystallin proteins (Jaenicke, 1999).

## Conclusion

The procedures generally used for hair shape modulation have negative effects on hairs' stiffness and physical properties. The development of new formulations to protect hair against the adverse effects of high temperature procedures is needed. In the present study, KP-Cryst Wt and KP-Cryst Mut proteins were successfully expressed, purified and applied on the pretreatment of virgin Asian and Caucasian Brown hair. Both proteins showed the capacity to bind and penetrate into the hair fibers, being their deposition or penetration pattern dependent on the formulation pH and hair type. For the Caucasian hair, the KP-Cryst proteins are localized preferentially at the hair surface at pH5, while at pH9 the proteins tend to penetrate through the inner layers of hair fiber, reaching the cortex. A similar behavior was observed for the Asian hair, however with a lower amount of protein inside the hair fiber, possibly due to the differences in the structure of the hair cuticles of this type of hair. A protective effect of KP-Cryst Wt and KP-Cryst Mut proteins was observed when both proteins were applied as a pretreatment before iron. The pretreatment with the fusion proteins preserved the mechanical properties and reduced the water loss for the Caucasian Brown hair after iron application.

This study provides new insights about the protective effect of KP-Crys Wt and KP-Cryst Mut proteins of hair fibers' when exposed to repeated procedures using high temperature. Both proteins are potential ingredients to be included in new hair thermal protection formulations.

## Data Availability Statement

The datasets generated for this study are available on request to the corresponding author.

## Author Contributions

AT performed the work on the expression and purification of KP-Cryst fusion proteins and performed the experiments for protein application on hair and its effects on hair properties and hair thermal protection. JG was responsible for BL21 transformation using specific vectors and optimization of the expression and purification conditions for both proteins. CS performed the MALDI-TOF measurements. AR worked in the design of KP-Cryst fusion proteins sequence and supervised all the work. AT, JG, CS, AC-P, and AR were equally responsible for manuscript drafting and editing. All authors revised the final version of the manuscript.

### Conflict of Interest

The authors declare that the research was conducted in the absence of any commercial or financial relationships that could be construed as a potential conflict of interest.
