# Supplementary material for: Crystallin Fusion Proteins Improve the Thermal Properties of Hair
Source: Front Bioeng Biotechnol. 2019 Oct 25;7:298. doi: 10.3389/fbioe.2019.00298 (PMC6823552; doi:10.3389/fbioe.2019.00298)
Supplement: Supplementary file 1 [file Data_Sheet_1.PDF]

Supplementary Material

1. Supplementary Figures

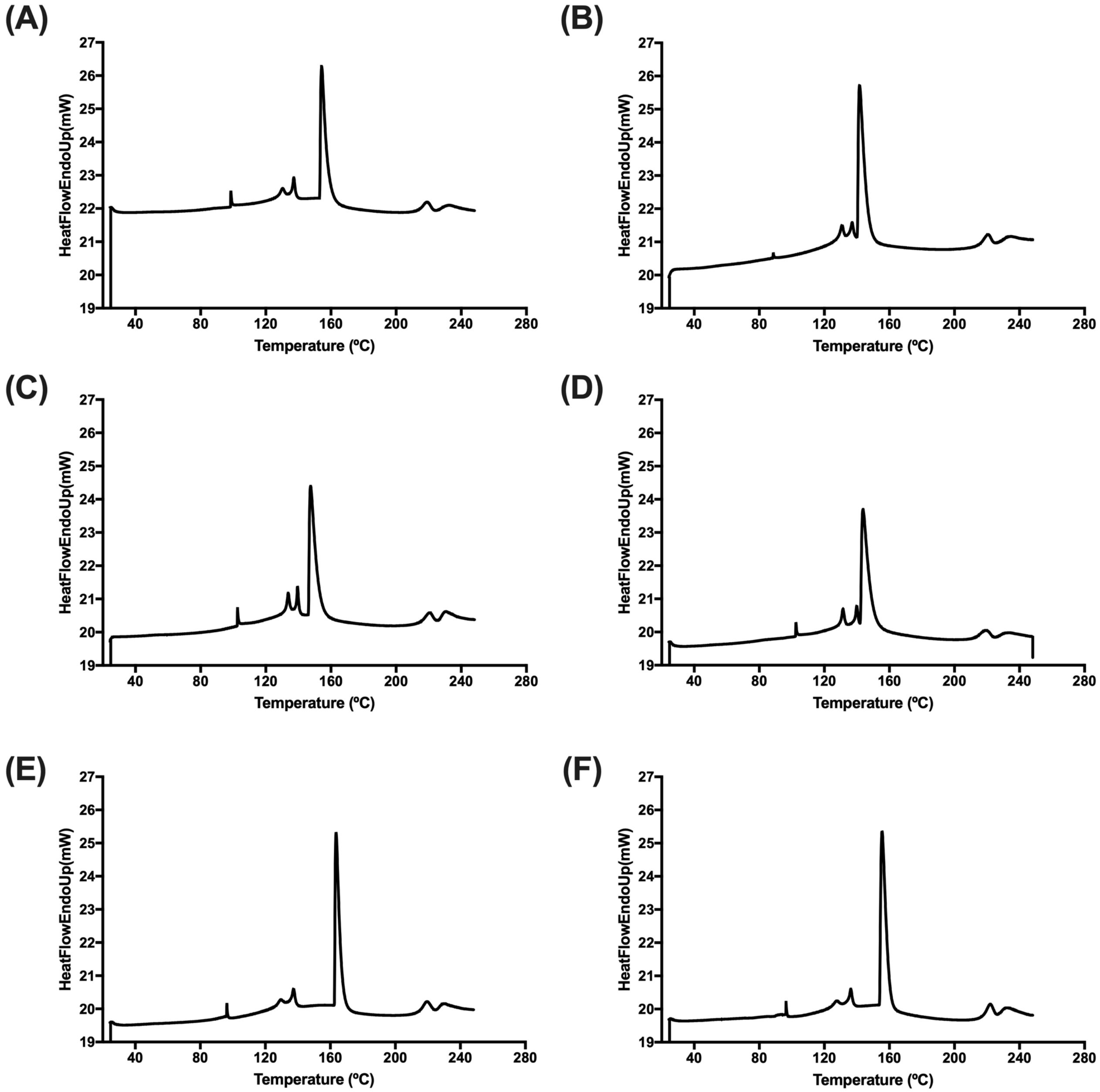

Supplementary Figure 1. DSC thermograms of Caucasian Brown hair treated with (A) buffer pH5, (B) buffer pH9, (C) KP-Cryst Wt pH5, (D) KP-Cryst Wt pH9, (E) KP-Cryst Mut pH5 and (F) KP-Cryst Mut pH9 proteins.

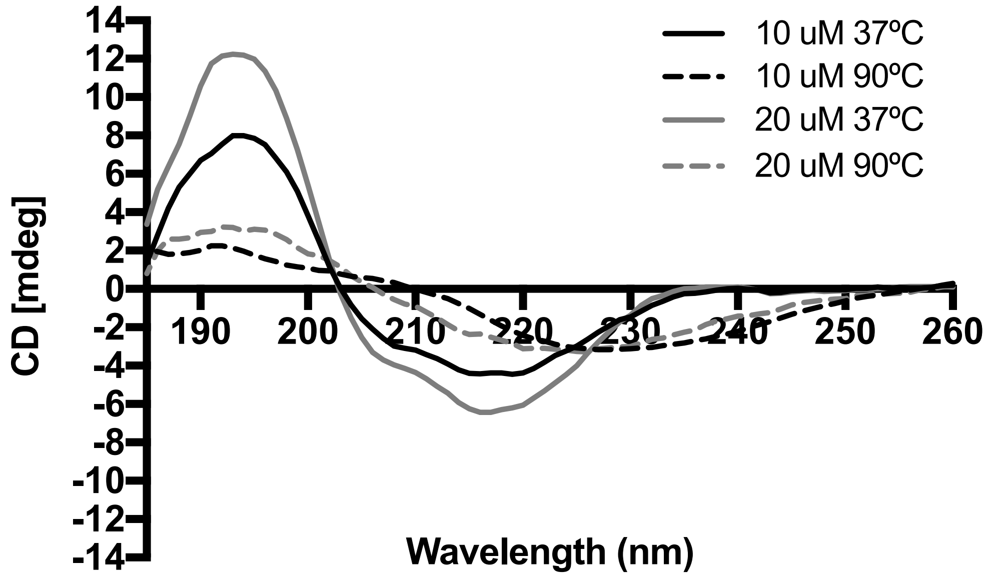

Supplementary Figure 2. Effect of protein concentration and temperature on the circular dichroism spectra of KP-Cryst Mut proteins. Experiments were conducted at 37 and 90 ºC.

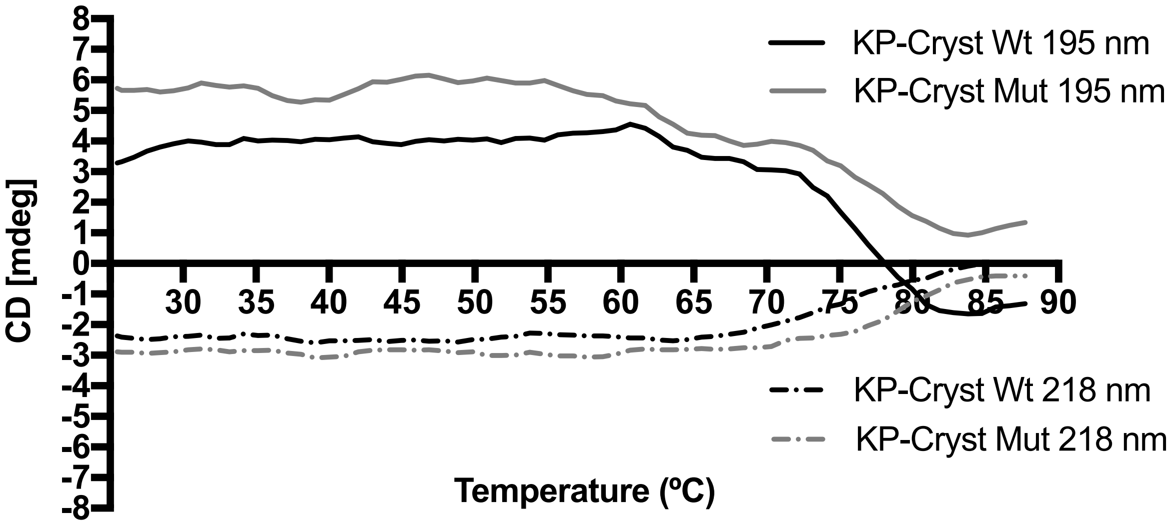

Supplementary Figure 3. Thermal unfolding curves obtained for KP-Cryst Wt and KP-Cryst Mut proteins by monitoring CD signal at 218 and 195 nm from 25 to 90ºC. All the spectra are background corrected.
